# Dual Doppler Effect in Wedge-Type Photonic Crystals

**DOI:** 10.1038/s41598-018-24941-8

**Published:** 2018-04-25

**Authors:** Qiang Jiang, Jiabi Chen, Liangcai Cao, Songlin Zhuang, Guofan Jin

**Affiliations:** 1 State Key Laboratory of Precision Measurement Technology and Instruments, Department of Precision Instruments, Tsinghua University, Beijing, 100084 China; 2 Optical Electronic Information and Computer Engineering College, University of Shanghai for Science and Technology, Shanghai, 200093 China

## Abstract

The dual Doppler effect, in the simultaneous occurrence of both normal and inverse Doppler effect in one moving two-dimensional wedge-type photonic crystal, is proposed. An improved finite-different time-domain algorithm is used to verify this phenomenon. The spatial Fourier Transformation has been applied to complex electrical field data to reveal the mechanism. The harmonics with negative spatial frequencies show a lagging phase evolution, while those with positive spatial frequencies show a front phase evolution. Different wedge-type photonic crystals are designed to filter out the required harmonics based on the systematic study of spatial Fourier Transformation and wave vector diagram. Our work paves a new way for Doppler cooling of atomic gases, radar deception, invisibility cloaks, microstructure dual frequency interferometer and so on.

## Introduction

The Doppler effect can be observed in every walk of life. Applications of the effect has been widely established, including radar, laser vibrometry, blood flow measurement, and the search for new astronomical objects. It can be described as the effect produced by a moving source of waves in which there is an apparent upward shift in the frequency for observers towards which the source is approaching. However, the inverse Doppler effect performs in an opposite way. In 1968, G. Veselago predicted that when electromagnetic waves propagated through materials with negative permittivity *ε* and negative permeability *μ*, the frequency should drop as a source moving towards an observer but increase as it moving away^1^. The inverse Doppler effect was first observed at radio frequency by N. Seddon with an experimental magnetic, nonlinear transmission line in 2003^2^. After that, this anomalous effect was observed in more than one kind of special dispersive media, like left-handed material (LHM) whose effective permittivity *ε* and permittivity *μ* are both negative, and photonic crystals (PhC) using shock discontinuities^3^.

LHM possesses the feature of negative refractive index (NRI)^4^. When a light travels through it, the phase velocity $\mathop{{v}_{p}}\limits^{\longrightarrow}$ is antiparallel to the Poynting vector $\vec{S}$. Interestingly, the NRI feature can also be observed in some PhCs whose equi-frequency contour (EFC) is shrinking towards center. Differing from the homogenous LHM, the NRI for the PhC is caused by complex dispersion of the periodic structure. The unusual dispersion leads to many extraordinary phenomena, such as self-collimation^5^, super-prism^6^, zero phase delay^7^, anomalous and negative refraction^8,9^. With one incident wave, the refracted wave in homogenous LHM is unique and acts as a backward wave, of which the energy flows forward while the equiphase front moves backward. Nevertheless, refracted light in the PhC will be no more unique. S. Foteinopoulou had made a detailed illustration how to determine the directions of all refracted lights in the PhC by using wave vector diagram^10^. Propagating waves in the PhC consist of both forward waves and backward waves^11,12^, which has been demonstrated experimentally by analyzing the phase evolutions for the retrieved harmonics of the measured Bloch wave^13^.

The inverse Doppler shift at optical frequency was observed in the PhC in our previous research in 2011^4^. Considering the explanations of N. Seddon’s experiment^2,14,15^, we have revealed that the inverse Doppler shift in the PhC is caused by some spatial harmonics in the PhC which have $\mathop{{v}_{g}}\limits^{\longrightarrow}\cdot \vec{k} < 0$ (the backward wave) in terms of the decomposition of Bloch waves, where $\mathop{{v}_{g}}\limits^{\longrightarrow}$ is the group velocity. As to the rest spatial harmonics which have $\mathop{{v}_{g}}\limits^{\longrightarrow}\cdot \vec{k} > 0$ (the forward wave), they cannot couple to the vacuum due to total inner reflection (TIR)^11^. Hence, it is significant to investigate the Doppler effect phenomenon in the PhC if both backward waves and forward waves couple to the vacuum by appropriate design of the wedge type of the photonic crystal.

In this work, by using the improved finite-different time-domain (FDTD) method, we utilize a moving wedge-type PhC to realize a novel phenomenon, dual Doppler effect, in which both normal Doppler shift and inverse Doppler shift occur simultaneously. For the purpose of illustrating the mechanism, we use spatial Fourier Transformation (SFT) on the complex electrical field data of Bloch wave in the propagation direction. Based on our developed algorithm, the phase evolutions of the spatial harmonics could be illustrated in a comprehensible way by the retrieved field and the wave vector diagram. The multiple outgoing waves from the slope of wedge-shape PhC could be determined in use of multiple reciprocal unit cells. It is firstly observed that the dual Doppler shift is governed by the forward wave and backward wave in the PhC, and the shifts can be calculated with the effective indexes of the PhC corresponding to each harmonic.

## Results

### Verification of the dual Doppler effect

**Figure 1:**
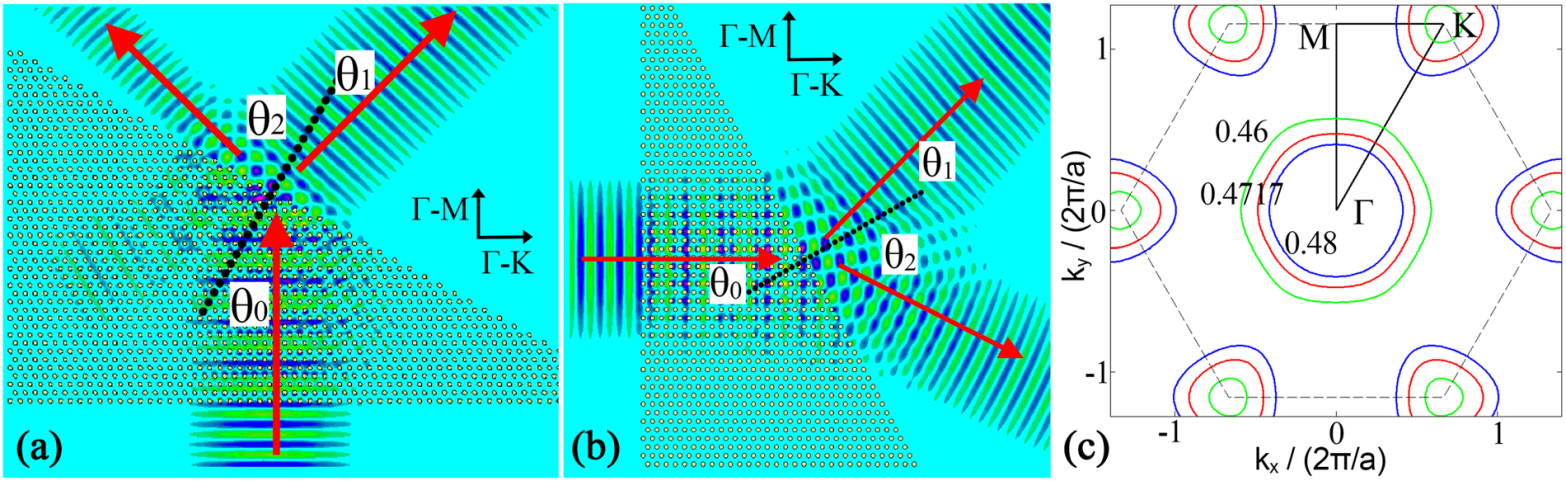
Two cases of waves exiting from the PhC along with positive refraction and negative refraction. (**a**,**b**) Wave propagation in two cases. (**c**) The EFC of the PhC shows a shrinking contour that the frequencies increase towards the Γ point.

The structure of the PhC under investigation is shown in Fig. 1, silicon rods with the radius of 0.226*a* (*a* is the lattice period) and *ε* of 11.96 form themselves as a wedge type^4,11^. This PhC, with the EFC showed in Fig. 1(c) portraying a shrinking contour at the normalized frequency of 0.4717(*2π/a*), has been verified experimentally to be a negative refraction material with the NRI measured as −0.5^4^. In that experiment, light travels along ΓM direction and the only light exits from the slope of which the vertex angle is 60°. Here, by changing the light propagation direction and the vertical angle, refracted light exiting from the slope can be divided into two beams. Figure 1 shows two cases, as for case 1 in Fig. 1(a), light traveling along ΓM direction (*y* direction) with the vertex angle of 30°, the silicon rods array with the period of $\sqrt{3}a$ forms the slope. While for case 2 in Fig. 1(b), light traveling along MK direction (*x* direction) with the vertex angle of 30°, the slope is formed by silicon rods array with the period of $a$.

**Figure 2:**
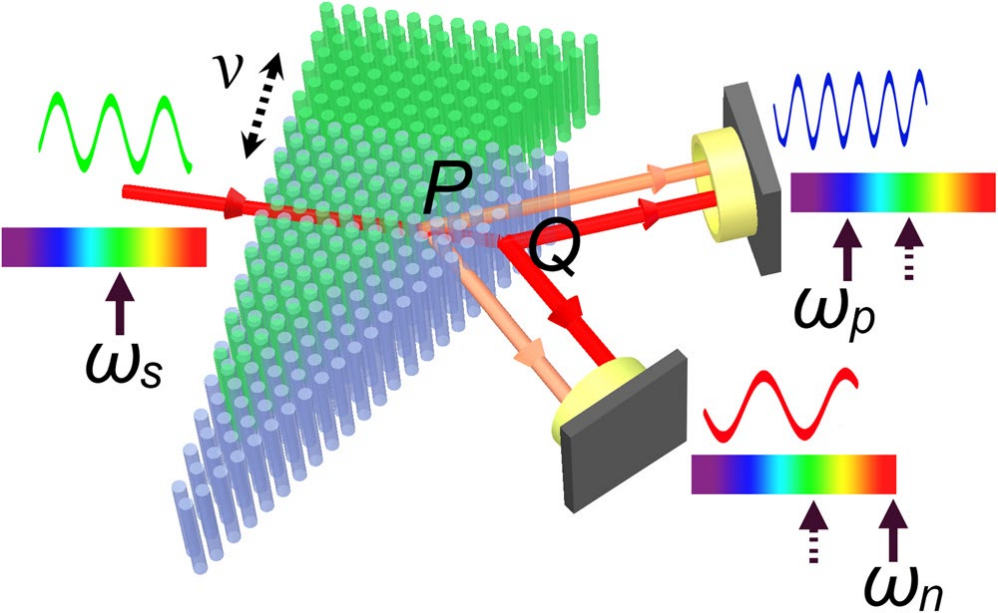
Sketch map of light traveling through the moving PhC prism. The detecting frequencies of outgoing lights varies in two different ways.

**Figure 3:**
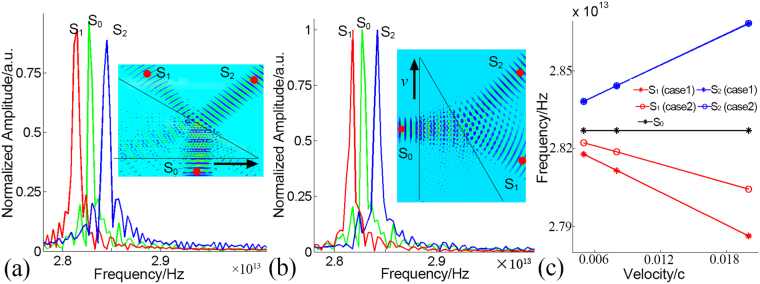
Doppler frequency shifts in these two cases. (**a**,**b**) Doppler frequency shifts of the refracted waves at velocity of 0.008*c* for two cases. The insets display the wave propagations in the moving PhCs, the red points represent the sampling points, bold black arrow shows the moving direction and black lines indicate the outline of the PhC. (**c**) Doppler frequency shifts of two refracted waves at velocities of 0.005*c*, 0.008*c* and 0.02*c*.

Refracted light beams in these two cases appear both positive and negative refractions. Since the negative refraction beam leads to inverse Doppler effect^4^, we speculate that with both positive and negative refractions happen simultaneously, a dual Doppler phenomenon will occur. We use the dynamic FDTD method mentioned above to verify that. Figure 2 shows the corresponding Sketch map, where the rods in green and purple indicate the PhC before and after moving, the double-sided arrow indicates the moving direction, the point P on the second interface of prism represents the position where the light exits and moves to Q with the motion of PhC. For the sake of saving running time and assuring the accuracy^11^, the moving speeds of the PhCs are set as 0.005*c*, 0.008*c* and 0.02*c*, where *c* is the speed of light in vacuum. With the PhCs moving at a speed of 0.008*c*, the corresponding field distributions are detected and exhibited in the insets of Fig. 3.

Three sampling points S_0_, S_1_ and S_2_, labeled with red dots in Fig. 3(a,b), are set to record electrical field of light source, negative refracted light and positive refracted light correspondingly. The corresponding time domain frequencies *ω*_*s*_, *ω*_*ni*_, and *ω*_*pi*_ (i = 1, 2, corresponding to case 1 and case 2) are then obtained by the fast Fourier Transformation (FFT) method. The spectrum shown in Fig. 3 manifests that the frequencies of positive refracted light and negative reflected light shift in opposite way, with their frequencies locating in both sides of the source frequency. As is shown in Fig. 3(c), for all the velocities, frequencies of negative refracted lights are larger than those of light source, while frequencies of positive refracted lights are smaller than the source frequency. Without loss of generality, the incident light in our model is chosen as CO_2_ laser, which possess a wavelength of 10.6 μm. The detecting source frequency *ω*_0_ is 2.827022 × 10^13^ Hz, which has a relative error of as low as −0.04% to the theoretical frequency *ω*_*s*_ of 2.82823 × 10^13^ Hz (calculated by *c/λ*). This confirms the accuracy of the dynamic FDTD algorithm.

### Analysis of the dual Doppler effect

**Figure 4:**
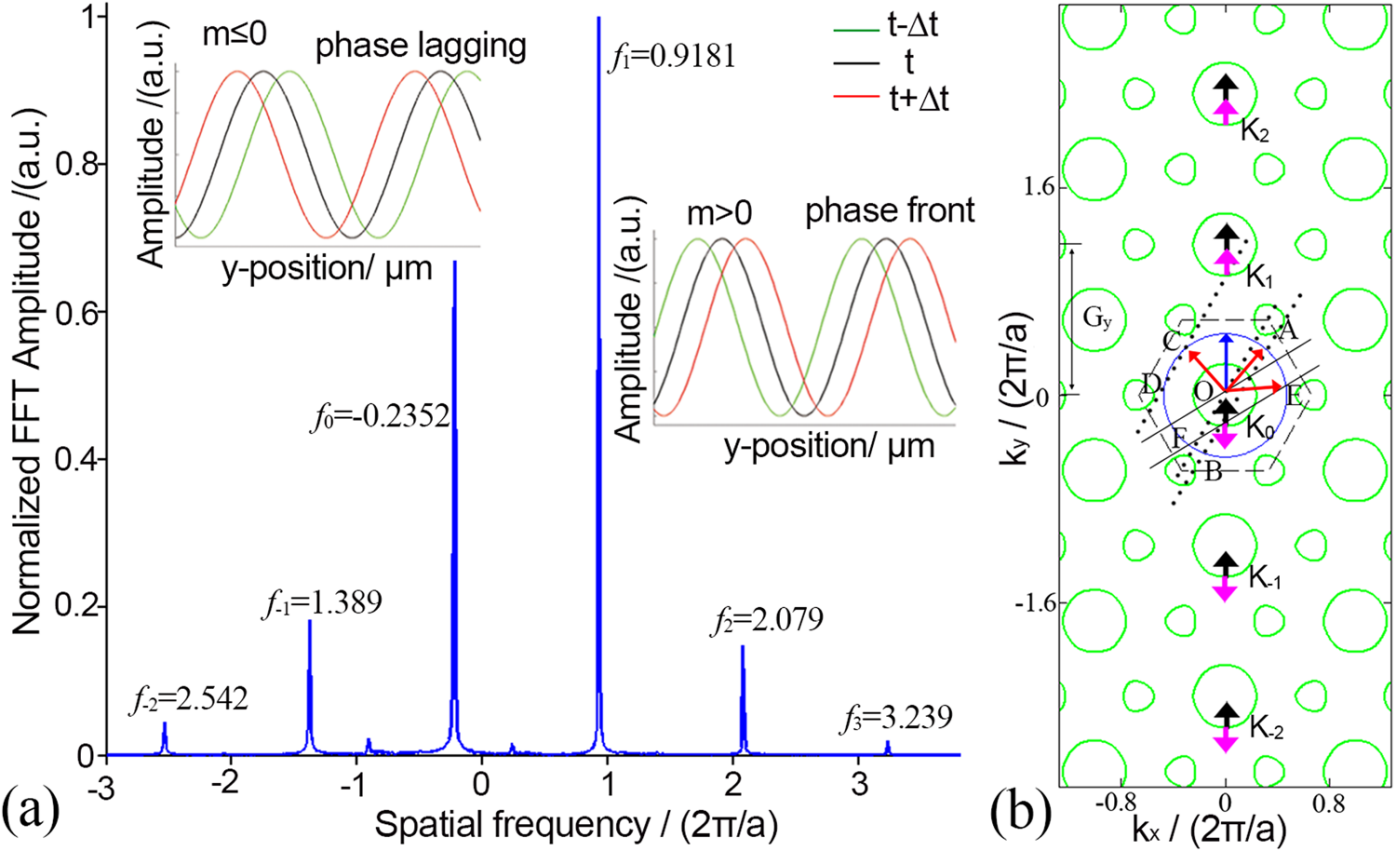
Spectra of the Bloch wave and the wave vector diagram along ΓM direction in case 1. (**a**) Spectra of the Bloch wave. The insets show phase evolutions of harmonics, in which the black line indicates the retrieved electrical field at one time, while the green line and red line indicate the fields of previous time and later time. (**b**) In reciprocal unit cells, the black arrows and pink arrows indicate the directions of Poynting vectors $\vec{S}$ and wave vectors $\vec{{K}_{m}}$ of each excited plane wave correspondingly. The red arrows indicate the correct exiting directions of refracted waves, i.e., OA and OC for case 1, OE for ref.^4^. The blue line shows the direction of incident wave from air.

To analyze the spatial harmonics in the PhC and identify the positive spatial frequencies and negative spatial frequencies, the SFT is applied for the complex electrical field along the propagation direction in the PhC^16^. As for case 1, the spatial spectrum for complex field data along ΓM direction, as shown in Fig. 4(a), has a series of peaks which have a certain periodic property. This can be illustrated from the perspective of Bloch theorem. Waves traveling in the PhC consist of a series of plane waves of which the wave vectors are periodic in respect to the reciprocal lattice vector. For conciseness, only several reciprocal unit cells are shown in Fig. 4(b), where the blue circle and green circles indicate the EFCs of air and PhC in the frequency of 0.4717*(2π/a)* correspondingly. With the phase match condition^17^, countless plane waves along ΓM direction are excited by the normal incident wave vector (bold blue arrow). Under the guidance of the principle that the Poynting vector should point to the shrinking direction and should be away from the source^10^, the directions of Poynting vectors $\mathop{{S}_{m}}\limits^{\longrightarrow}$ and the wave vectors $\mathop{{K}_{m}}\limits^{\longrightarrow}$ (m is the order, m $\in {\mathbb{Z}}$) of the excited plane waves can be determined, as shown in Fig. 4(b), labeled with black and pink arrows respectively. Wave vectors $\mathop{{K}_{m}}\limits^{\longrightarrow}$ can be written as $\mathop{{K}_{m}}\limits^{\longrightarrow}=\mathop{{K}_{0}}\limits^{\longrightarrow}+m\cdot \mathop{{G}_{y}}\limits^{\longrightarrow}$, where $\mathop{{K}_{0}}\limits^{\longrightarrow}$ is the wave vector in first Brillouin zone (BZ), $\mathop{{G}_{y}}\limits^{\longrightarrow}$ is the reciprocal lattice vector (4π/(√3*a*)^18^). Some excited waves are forward waves when $\mathop{{K}_{m}}\limits^{\longrightarrow}\cdot \mathop{{S}_{m}}\limits^{\longrightarrow} > 0$, while other excited waves are backward waves when $\mathop{{K}_{m}}\limits^{\longrightarrow}\cdot \mathop{{S}_{m}}\limits^{\longrightarrow} < 0$. The spatial frequencies for the spatial harmonics in Fig. 4(a) can be written as ${f}_{m}=1/{\lambda }_{m}$. Hence, on the quantitative relation, ${f}_{m}$ can be expressed as ${f}_{m}={f}_{0}+m\cdot {G}_{y}/2\pi$. This periodicity is obvious in the spectra of Fig. 4(a). Furthermore, the phase evolution for each spatial harmonics is obtained by using FFT and iFFT methods^7^, as is shown in the inset of Fig. 4(a). It shows the phase of harmonics with *m* ≤ 0 is lagging, which corresponds to the backward waves. While for harmonics with *m* > 0, the phase is front, which corresponds to forward waves. These are consistent with the analysis in the wave vector diagram in Fig. 4(b).

For each spatial harmonic, the corresponding effective refractive index of the PhC can be assigned as $n={f}_{m}\,/{f}_{s}$, where *f*_*s*_ = 0.4717/*a* is the spatial frequency of light source. Considering the TIR condition, with regard to the reflection happened on the slope of the PhC, only the corresponding spatial harmonics with a corresponding |*n*| smaller than 1/sin(θ_0_) can couple into the vacuum. Among all these harmonics, only *f*_0_ (n = −0.498) and *f*_1_ (n = 1.946) are satisfactory, with the emergent angles being 0.08π and 0.43*π* in terms of Snell’s law. Actually, the outgoing directions of reflected lights can also be predicted precisely from the wave vector diagram. As is shown in Fig. 4(b), the dotted lines represent the normals of the interface, also known as the conservation lines. The conservation line of *K*_0_ intersects the EFC of air at two points A and B, while another line for *K*_1_ intersects the EFC of air at points C and D. In consideration of the causality, the correct directions of the outgoing waves can be determined as OA and OC, as labeled by red arrows, which agrees with the propagation in Fig. 1(a). As for other plane waves, the corresponding conservation lines have no intersection with EFC of air, which means they cannot couple to the vacuum. Likewise, the only refractive light for our previous work^3^ is also clarified in Fig. 4(b). As the conservation line (represented by the solid black line) intersects the EFC of air at points E and F. As expected, only OE is the right emergent direction.

**Figure 5:**
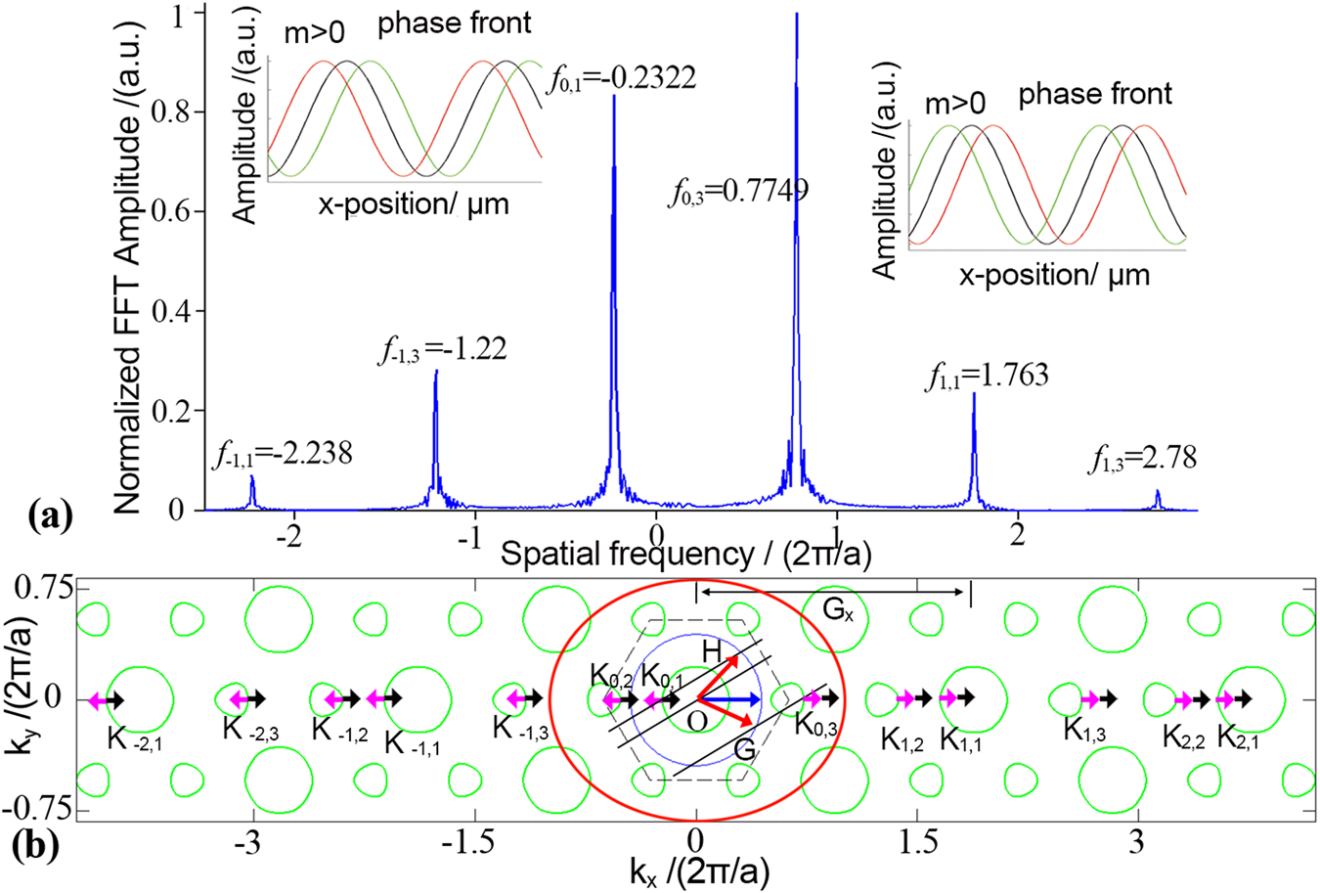
Spectra of the Bloch wave and the wave vector diagram along MK direction in case 2. (**a**) Spectra of the Bloch wave. Peaks here can be divided into three groups in terms of the order m, each group has two peaks, *f*_*m*,*1*_ and *f*_*m*,*3*_. The insets show the phase evolution of the retrieved electrical fields. (**b**) In reciprocal unit cells, the solid lines and dash lines represent the conservation lines. The red arrows indicate the correct exiting directions of refracted waves, i.e., OG and OH.

In case 2, the Bloch waves traveling along MK direction possess a period of *G*_*x*_ = 4π/*a*^18^. Wave vector diagram in Fig. 5(b) shows three excited plane waves in one reciprocal unit (as shown in the red circle). Hence, the corresponding wave vectors can be expressed as $\mathop{{K}_{m,i}}\limits^{\longrightarrow}=\mathop{{K}_{0,i}}\limits^{\longrightarrow}+m\cdot \mathop{{G}_{x}}\limits^{\longrightarrow}$, where $\mathop{{K}_{0,i}}\limits^{\longrightarrow}$ is the wave vector in first reciprocal unit, and *i* = 1, 2, 3 represent those three excited waves. Likewise, the spatial frequencies in the spectra can be expressed as ${f}_{m,i}={f}_{0,i}+m\cdot {G}_{x}/2\pi$. However, the components of *f*_*m*,*2*_ vanish in the spectra. A possible explanation can be given from the perspective of the definition of group velocity, ${v}_{g}={\nabla }_{k}\omega$. That the equi-frequency contour is sharp when the *f*_*m*,*2*_ components are excited, which makes the derivative for wave vector of the sharp point discontinuous. Thus, it leads a failure to define a certain direction of group velocity. Nonetheless, peaks in Fig. 5(a) can be divided into three groups from the perspective of order number *m*, with a spatial interval of ${G}_{x}/2\pi$. Phase evolutions for these harmonics depicted in the inset of Fig. 5(a) show that the harmonics are forward for *m* > *0* while backward for *m* ≤ *0*. Similarly, the directions of outgoing light beams can also be easily determined from the wave vector diagram. As is shown in Fig. 5(b), two conservation lines intersect the EFC of air at four points. To preserve causality, only OH and OG point to the reasonable direction. The exact angles can be obtained from the spectra with the method mentioned above, as *θ*_1_ of 14.18° for negative refraction and *θ*_2_ of 54.48° for positive refraction.

Waves traveling in the PhC consist of both forward waves and backward waves in these two cases. Taking one of them to analyze the Doppler effect, as showed in Fig. 2, the PhC prism moves with a velocity of *v* while light traveling through it. Therefore, the Doppler frequency shift *ω*′ at the second interface can be calculated simply as^4^

1 $${\rm{\omega }}^{\prime} ={\omega }_{s}(1-nv\cdot \,\tan \,{\theta }_{0}/c),$$

In the proposed PhC, the refractive index *n* can be either positive or negative, which makes the Doppler effect diversified, supporting the dual Doppler effect. The relative movement between the exiting point of light on the slope and the static detecting surface (as shown by the blue bold line) causes the second Doppler effect. Finally, the received frequencies of the outgoing negative refractive and positive refractive beam can be calculated by

2 $${{\rm{\omega }}}_{cn}={\omega }_{s}\frac{c-{n}_{1}v\cdot {\tan {\rm{\theta }}}_{0}}{c-v\cdot {\tan {\rm{\theta }}}_{0}\,\cos ({{\rm{\theta }}}_{1}+{{\rm{\theta }}}_{0}))},$$

3 $${{\rm{\omega }}}_{cp}={\omega }_{s}\frac{c-{n}_{2}v\cdot {\tan {\rm{\theta }}}_{0}}{c-v\cdot {\tan {\rm{\theta }}}_{0}\,\cos ({{\rm{\theta }}}_{2}-{{\rm{\theta }}}_{0}))},$$

where *n*_1_ and *n*_2_ are effective refractive indexes of the PhC corresponding to those two spatial harmonics which show negative refraction and positive refraction. The calculated dual Doppler frequencies (*ω*_*cn*_, *ω*_*cp*_) and the detected values (*ω*_*n*_, *ω*_*p*_) for those three velocities are showed in Table 1.

**Table 1:** Detecting and theoretical values for dual Doppler effect.

	Velocities	Frequencies at point S_1_/× 10^13^ Hz			Frequencies at point S_2_/× 10^13^ Hz		
		*ω*_*cn*_	*ω*_*n*_	Relative errors	*ω*_*cp*_	*ω*_*p*_	Relative errors
Case 1	0.005*c*	2.83922	2.83816	0.037%	2.81787	2.8178	0.002%
	0.008*c*	2.84532	2.84414	0.042%	2.81482	2.81153	0.117%
	0.02*c*	2.87026	2.86819	0.072%	2.79168	2.78627	0.19%
Case 2	0.005*c*	2.83617	2.83821	−0.072%	2.820923	2.8223	−0.049%
	0.008*c*	2.84227	2.84422	−0.069%	2.817873	2.81873	−0.03%
	0.02*c*	2.86362	2.86841	−0.167%	2.79958	2.80432	−0.169%

The Doppler frequencies deduced from equation (2) and equation (3) are verified precisely by the dynamic FDTD method with a relative error of less than 0.2% between detecting values and theoretical values. With ${\omega }_{n} > {\omega }_{s}$ and ${\omega }_{p} < {\omega }_{s}$, the dual Doppler effect that both inverse Doppler effect and normal effect appeared simultaneously are proved successfully.

## Discussion

In conclusion, we have found a novel phenomenon, the dual Doppler effect, with both normal and inverse Doppler effect occurring simultaneously in one moving two-dimensional wedge type PhC. It was obtained by a dynamic two-dimensional FDTD method which can deal with the situation of electromagnetic wave propagation in moving objects. The theoretical values showed a high consistence with the detecting values along with a relative error less than 0.2%. In addition, we have shown that the outgoing direction of the complex harmonic coupling to the vacuum can be predicted precisely by using multiple reciprocal unit cells. The novel phenomenon could benefit any technique currently exploiting the conventional Doppler effect, such as the Doppler cooling of atomic gases, radar deception, invisibility cloaks, microstructure dual frequency interferometer^19,20^ and so on. The technique is also applicable to systems with tunable sources of terahertz radiation and other frequency regions that are difficult to access.

## Methods

### Spatial Fourier Transform

The obtained optical field in the structure is a complex field A·exp(*iφ*). A complex Spatial Fourier Transformation (SFT) is used to retrieve both the wavevector (*k*) and amplitude (A) of the field propagating. The SFT of the complex optical field also reveals the sign of k, i.e. both positive and negative wavevectors. As shown in the following equation, the convolution operation shifts the spatial frequencies to the right positions along the axis in the spectrum.

$$ {\mathcal F} ({\rm{A}}\cdot \exp (i\phi ))= {\mathcal F} ({\rm A})\ast  {\mathcal F} (\exp (i\phi ))= {\mathcal F} ({\rm{A}})\ast {\rm{\delta }}({k}_{x}-\frac{\phi }{2\pi x}).$$

### Dynamic FDTD algorithm

**Figure 6:**
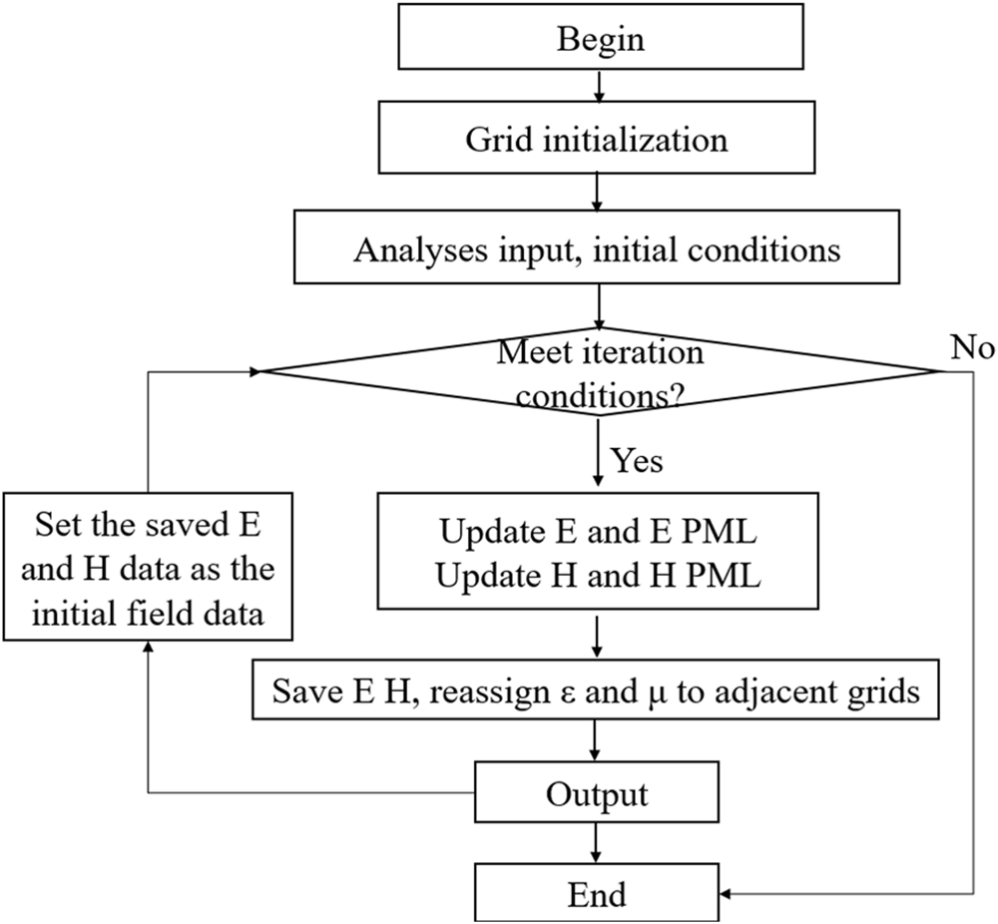
Flow chart of the improved FDTD algorithm. The distribution of ε and μ changes during each iteration.

To investigate the performance of the Doppler effect, a dynamic algorithm is developed based on the traditional FDTD method. In the general FDTD algorithm, the position of object is represented by assigning special ε and μ to the corresponding grids. It cannot be changed during the whole iterative operation, making it useless in dealing with the case of wave propagation in a moving object. The dynamic FDTD method can solve this problem. As shown in Fig. 6, the improved FDTD algorithm can be simply described as: the parameters of ε and μ are reassigned to the adjacent space grids before each iterative operation, so as to reappear the movement of the object. For example, the object moves in a speed of *v*, the time step is *Δt* and the grid size is *Δx*, then the parameters of ε and μ will be reassign to the grids v*Δt/Δx* away from the present girds. Before each reassigned operation, the electromagnetic field data have been saved, and after the reassignment, the saved field data is used as the initial field data for the coming iterative operation.

## References

[1] Veslago V (1968). The electrodynamics of substances with simultaneously negative values of ε and μ. Sov. Phys. Usp..

[2] Seddon N, Bearpark T (2003). Observation of the inverse Doppler effect. Science.

[3] Reed E, Soljačić M, Joannopoulos J (2003). Reversed Doppler Effect in Photonic Crystals. Phys. Rev. Lett..

[4] Chen J (2011). Observation of the inverse Doppler effect in negative-index materials at optical frequencies. Nature Photon..

[5] Kosaka H (1999). Self-collimating phenomena in photonic crystals. Appl. Phys. Lett..

[6] Kosaka H (1999). Superprism phenomena in photonic crystals. Phys. Rev. B.

[7] Kocaman S (2011). Zero phase delay in negative-refractive-index photonic crystal superlattices. nature Photon..

[8] Gralak B, Enoch S, Tayeb G (2000). Anomalous refractive properties of photonic crystals. J. Opt. Soc. Am. A.

[9] Notomi M (2000). Theory of light propagation in strongly modulated photonic crystals: Refractionlike behavior in the vicinity of the photonic band gap. Phys. Rev. B.

[10] Foteinopoulou S, Soukoulis CM (2005). Electromagnetic wave propagation in two-dimensional photonic crystals: A study of anomalous refractive effects. Phys. Rev. B.

[11] Jiang Q (2016). Mechanism Analysis of the Inverse Doppler Effect in Two-Dimensional Photonic Crystal based on PhaseEvolution. Scientific reports.

[12] Martínez A, José Sánchez-Dehesa HM, Martí J (2005). Analysis of wave propagation in a two-dimensional photonic crystal with negative index of refraction-plane wave decomposition of the bloch modes. Opt. Express.

[13] Gersen H (2005). Direct Observation of Bloch Harmonics and Negative Phase Velocity in Photonic Crystal Waveguides. Phys. Rev. Lett..

[14] Kozyrev AB, van der Weide DW (2005). Explanation of the Inverse Doppler Effect Observed in Nonlinear Transmission Lines. Phys. Rev. Lett..

[15] Reed EJ, Soljacic M, Ibanescu M, Joannopoulos JD (2004). Comment on “Observation of the inverse Doppler effect”. Science.

[16] Engelen RJP (2005). Local probing of Bloch mode dispersion in a photonic crystal waveguide. Opt. Epress.

[17] Jackson, J. D. Classical electrodynamics (Wiley Publications, 1999).

[18] Joannopoulos, J. D., Johnson, S. G., Winn, J. N. & Meade, R. N. Photonic crystals: molding the flow of light (Princeton university press Published, 2011).

[19] Zhu Z (2017). A Method for Measurement of Nonlinearity of Laser Interferometer Based on Optical Frequency Tuning. Sensor.

[20] Merzouk WA (2016). Highly compact and easy-to-use optical chip interferometer with picometric performances. Review of Scientific Instruments,.

